# Antibacterial agent-releasing scaffolds in dental tissue engineering

**DOI:** 10.34172/japid.2021.003

**Published:** 2021-03-31

**Authors:** Zahra Gholami, Shirin Hasanpour, Samira Sadigh, Sana Johari, Zahra Shahveghar, Kosar Ataei, Eelahe Javari, Mahsa Amani, Leila Javadi Kia, Zahra Delir Akbari, Zahra Nazari, Solmaz Maleki Dizaj, Yashar Rezaei

**Affiliations:** ^1^Faculty of Dentistry, Tabriz University of Medical Sciences, Tabriz, Iran; ^2^Dental and Periodontal Research Center, Tabriz University of Medical Sciences, Tabriz, Iran

**Keywords:** Antimicrobial agents, Endodontics, Oral diseases, Pediatric dentistry, Periodontitis, Scaffolds, Stem cell, Tissue engineering

## Abstract

It seems quite challenging in tissue engineering to synthesize a base material with a range of essential activities, including biocompatibility, nontoxicity, and antimicrobial activities. Various types of materials are synthesized to solve the problem. This study aimed to provide the latest relevant information for practitioners about antibacterial scaffolds in dental tissue engineering. The PubMed search engine was used to review the relevant studies with a combination of the following terms as search queries: tissue engineering, scaffolds, antimicrobial, dentistry, dental stem cells, and oral diseases. It is noteworthy to state that only the terms related to tissue engineering in dentistry were considered. The antimicrobial scaffolds support the local tissue regeneration and prevent adverse inflammatory reactions; however, not all scaffolds have such positive characteristics. To resolve this potential defect, different antimicrobial agents are used during the synthesis process. Innovative methods in guided tissue engineering are actively working towards new ways to control oral and periodontal diseases.

## Introduction

The science of tissue engineering has excellent potential for tooth regeneration. There are three essential elements in tissue engineering: scaffolds, stem cells (SCs), and growth factors (GFs).^1,2^ Scaffolds and growth factor carriers are important players in the regeneration of damaged tissues or teeth.^3^

Tissue engineering requires different biomaterials with distinct properties than the ones used in engineering other tissues. Dental tissue engineering deals with regenerating damaged or lost tooth components, including enamel, dentin, and pulp. Postnatal investigation of the tooth development process is a crucial step for identifying the factors affecting the regeneration of dental tissues.^4,5^ As a complex tissue, a tooth consists of hard tissues, dentin, and enamel and is connected to bone through ligaments. Successive and mutual interactions between epithelial-mesenchymal cells shape teeth. While the epithelial cells have a prominent role in enamel formation, mesenchymal cells are responsible for producing differentiated cells vital for the formation of odontoblasts, pulp, and periodontal ligament.^6,7^

Tissue engineering makes use of a wide range of different materials: hydroxyapatite, various composites based on bioactive glass, and synthetic/natural polymers. In addition, it is also possible to use 3D printing technology for scaffold production.^8^ None of the options mentioned above for bone and dental tissue engineering match all the characteristics of bone graft substitutes.^9^ Another instance would be electrospinning, which is a practical technique since its versatility allows for the synthesis of micro- and nano-fibers. Notably, one favorable characteristic of these nano-fibers is their optimum flexibility in the fabrication process, but low hydrophilicity and having no surface cell-recognition sites lead to sub-optimal performance of synthetic materials. Conversely, natural fibers have optimal biocompatibility performance, but mechanical performance is their Achilles heel. The final comprehensive solution to meet both mechanical and biocompatible (bioactive surface) requirements is to develop composite fibrous scaffolds, in which the synthetic polymers serve as the backbone and the natural polymers provide cellular attachment.^10^

The main challenge after implementing scaffolds is to formulate strategies to prevent chronic infection after implantation,^11^ which is a possible complication expected in almost every surgical procedure. The culturing and implantation are the stages in which contamination is more likely. To combat contamination, antibiotics are an obvious solution, as they can effectively prevent bacterial infection after artificial bone transplantation. Typically, antibiotics should be prescribed with care since misuse leads to an adverse phenomenon: drug resistance.^12^ As a result, more effective but safer alternatives must be developed, and antibacterial scaffolds can be the ideal alternative. Standard antimicrobial scaffolds support local tissue regeneration and prevent adverse inflammatory reactions. Reportedly, in the synthesis of antibacterial scaffolds, different particles with optimal antibacterial and minimal toxic properties, such as silver nanoparticles (Ag), are used.^13^ This paper provides the latest information for practitioners about the antibacterial scaffolds in dental tissue engineering.

### Antimicrobial agents in dental tissue engineering

The bacterial infections of the dental and periodontal defects should be managed and eradicated.^14^ Many antibiotics are embedded in polymer membranes, including but not restricted to tetracycline hydrochloride, metronidazole, and amoxicillin.^15^ Dayaghi et al^16^ compared the antimicrobial activity of Mg-Zn scaffolds containing a high tetracycline concentration and reported that this new scaffold has significant activity against *Staphylococcus aureus* and *Escherichia coli* compared to the reference scaffold mentioned above. Because of the significant antibacterial activity, if the tetracycline percentage is between 1% and 5%, it is a potential choice for bone healing applications. However, due to ever-increasing antibiotic resistance, different alternative substances have also been proposed as antimicrobial agents in dental tissue engineering, including metal and metal oxides, medicinal plants (herbal medicines), polymers, and novel drug delivery systems (nano-biomaterials).

#### Metals and metal oxides

The macromolecular construction suggests a tool to improve antimicrobial activity as numerous antimicrobial moieties, such as metals and metal oxides, can be conjugated to the scaffold. New studies and tests on gram-positive and gram-negative bacteria have demonstrated that metal-based antimicrobial macromolecules can effectively prevent and treat infections due to resistant strains.^17^

##### Silver (Ag)

Silver nanoparticles have attracted considerable attention due to their antibacterial effects on gram-positive and gram-negative bacteria, particularly multidrug-resistant strains, and their low toxic effects.^17^ Xing et al^18^ compared the antimicrobial activity of metallic silver particle-loaded poly-(3-hydroxybutyrate-co-3-hydroxyvalerate) (PHBV) scaffolds and poly-(3-hydroxybutyrate-co-3-hydroxyvalerate) and reported the significant antibacterial activity of Ag-loaded scaffold against *S. aureus* and *Klebsiella pneumonia* compared to the free PHBV scaffold. Numerous studies have incorporated Ag into scaffolds for sustained release and antibacterial activity. Tests on the antimicrobial activity of Ag scaffolds against *S. aureus* and *E. coli* showed that the minimum inhibitory concentration (MIC) and minimum bactericidal concentration (MBC) values for each strain were 32.0 and 32.0 μg/mL for *S. aureus*, and 64.0 and 85.3 μg/mL for *E. coli*. The control scaffold (without Ag) did not exhibit any antimicrobial effects.^19^

##### TiO_2_

In another study, TiO_2_-loaded scaffolds exhibited significant antibacterial activity. TiO_2_ might cause oxidative and mechanical damage to bacteria through contact activities and the production of reactive oxygen species (ROS).^20^ ROS exerts oxidative stress on bacteria while the concentration is higher than the bacterial antioxidant defense system’s inhibitory potential, thereby destroying the organization and action of bacteria.^21^ In addition, the contact between the bacterial cell wall and TiO_2_ leads to mechanical stresses and distortion of the bacterial cell membrane.^22^

#### Medicinal plants (herbal medicines)

##### Aloe vera

According to reports, the aloe vera plant, its bioactive components, and glucomannan (known as acemannan) have antiviral and bactericidal effects.^23^ It has been reported that these plants exhibit potent anti-inflammatory, antioxidant, and antibacterial activities due to their anthraquinones (e.g., barbaloin, emodin, and anthranol) and phenolic compounds.^24^ In addition to the antimicrobial activity of aloe vera, positive interactions with dental cells make it an appropriate candidate for periodontal therapy. It has been reported that aloe vera has antimicrobial activity against the primary bacteria associated with periodontitis. The gel of aloe vera has been shown to prevent the growth of both gram-negative and gram-positive bacteria compared to conventional antibiotics, such as vancomycin, methicillin, bacitracin, and erythromycin.^25^

##### Manuka honey

Manuka honey (MH) contains a unique Manuka factor, providing a supplementary antibacterial agent. The effect of MH-containing scaffold (hydrogel type) was investigated on bacterial elimination, adhesion, and cellular adhesion. The results showed higher antimicrobial activity for MH-containing scaffolds compared to conventional scaffolds.^26^

##### Berberine

Berberine acts as a natural antimicrobial agent and is active against many different bacteria, without toxic effects on mammalian cells. Huang et al^27^ prepared berberine-loaded scaffolds that showed more than 150 hours of berberine release, with potent antibacterial activity against *S. aureus*.

##### Curcumin

Numerous reports demonstrated the antibacterial, antiviral, antifungal, and antimalarial activities of curcumin.^28,29^ Based on recent studies, curcumin inhibits the growth and proliferation of *E. coli* at a concentration of 8 mg/mL.^30^ Besides, curcumin can prevent the growth of different methicillin-resistant *S. aureus* strains at concentrations of 125-250 mg/mL.^31^ Previous studies have also shown that local use of curcumin-containing scaffolds decreases gingival inflammation.^31-33^ Moreover, curcumin can efficiently inhibit the activation of inflammatory mediators and positively impact periodontal diseases.^31,32^

#### Polymers

##### Chitosan

Chitosan is a well-known scaffold in different tissue engineering fields. It has non-toxic degradation products with little effect due to its low hydrophilicity and low cell compatibility. It also has excellent antimicrobial activity against different bacteria. Besides, chitosan monomers support the regeneration of dental pulp wounds and are useful scaffolds for dental pulp cells.^34,35^

#### Novel drug delivery systems (nanobiomaterials)

Extensive data have shown that antibiotic pastes and chemical irritants might influence dental stem cells’ viability and function.^36^ In this case, a biocompatible intracanal drug delivery device based on nano-fibers is suggested to establish a bacteria-free atmosphere conducive to tissue restoration.^37^ In summary, a polymer-loaded solution must be formulated with the selected antibiotic(s) at the desired concentration.^38^ After this, antibiotic eluting nano-fibers are developed by modifying the electrospinning variables (e.g., flow rate, power of the field, etc.). These therapeutic nano-fibers could be conveniently inserted into the necrotic dental root canal system as a three-dimensional (3D) tubular structure, with excellent clinical potential since it will ensure that the antibiotics are distributed on microbial biofilms.^37^ The contaminated dentin subjected to triple-antibiotic-eluting nano-fibers showed remarkable bacterial mortality based on data from confocal laser scanning microscopy.^38^

## The use of antibacterial agent-releasing scaffolds in dental tissue engineering

### Endodontics and pediatric dentistry

The clinical treatment of premature (open apex) teeth with diseased pulp induced by trauma or bacterial infection is a concern for endodontists and pediatric dentists.^39^ The favorable treatment has, over the years, been compatible with the concepts of apexification, i.e., calcium hydroxide disinfection accompanied by root canal obturation using gutta-percha. However, fresh dental pulp recovery opportunities due to the evoked bleeding (EB) were raised during the past decade.^40^ However, the patient-dependent reconstruction process’s outcome is still unattainable and somewhat uncertain, despite the cases described in the clinical and histological analyses.^40^ Several factors were assumed to account for the unspecific success, including but not restricted to using rather cytotoxic antibiotic pastes.^36^ Several studies^3,3–20^ have reported the application and transmission prospects of 3D nano-fibers appropriate for antibiotic removal as a local technique for interior drug delivery that combined with injectable scaffolds, enriched or not with stem cells and growth factors (GFs), can increase the likelihood of the restoration of the human dental pulp.^36^

#### Periodontitis

Periodontitis is one of the most aggressive recurrent oral inflammatory disorders and damages soft and hard tissue consistency, resulting in tooth loss in severe tissue destruction cases.^41^ To restore the periodontal system’s architecture and function, in principle, targeted procedures of tissue reconstruction are used. In summary, an occlusive biocompatible polymer-based membrane is effectively used as a barrier to prevent the movement of epithelial and connective tissue cells to the regenerating site. Thus, smaller migrating ancestral cells in the residual periodontal ligament (PDL) can increase the root region’s porosity such that they can be differentiated into new periodontal tissues.^42^ The last decade has witnessed substantial improvements in the production of membranes with antimicrobial benefits with varying clinical success rates using this strategy. The works reported in the literature have included antimicrobials and inorganic particles (e.g., calcium phosphates) and biomolecules (e.g., growth factors) in the fabrication of membranes with therapeutic functions.^43^ More recently, combining known materials and biomolecules with advanced technologies has enabled the management of significant periodontal disorders.^44^ 3D printing has also been used for the first patient-specific growth factor adjusted scaffold (rhPDGF-BB).^45^

In recent years, novel treatments for dental pulp restoration, including the EB procedure, have been promising to improve treatment outcomes. During EB, after thorough root canal disinfection, periapical tissue laceration is purposely undertaken to induce bleeding and establish a fibrin-based scaffold to interfere with innate stem cells and growth factors. To disinfect, the EB procedure has ideally used a triple (ciprofloxacin/CIP, metronidazole/MET, and minocycline/MINO) or double (minocycline-free) antimicrobial ingredient made of very concentrated antibiotic pastes. Nevertheless, no therapeutic dosage was found to improve antibiotic mixture-specific antimicrobial activity, decreasing the host tissue and cell toxicity. Regardless of EB’s promising results in treating immature permanent teeth with necrotic pulps,^46^ one case study showed pulp-like tissue development.^47^ Evidently, most histological observations suggest that periapical tissue comprising bone-like hard tissue and cement-like content has been invaginated, contributing to the thickening of root canal walls.^48^ While the EB approach was suggested for treating immature teeth, a new survey showed that undifferentiated MSCs had reached the pulpal area of mature teeth with apical defects from the apical area.^49^

## Conclusion

New methods in dental tissue engineering have been employed towards new ways of managing oral and periodontal diseases. Antimicrobial agent-containing scaffolds in dental tissue engineering not only can support the local tissue regeneration but also can prevent adverse local inflammatory processes. Despite the currently available studies, more in vitro and in vivo studies are necessary in this field.

## Authors’ contributions

The study was planned by SMD. Data collection was supported by ZG, SH, SS, SJ, ZS, KA, EJ, MA, LJ, ZD, and ZN. SMD and YR contributed to the drafting and revision of the manuscript. SMD and YR are the corresponding authors of the manuscript. All authors read and approved the final manuscript.

## Ethics approval

Not applicable.

## Competing interests

The authors declare that they have no competing interests with regards to authorship and/or publication of this paper.
